# Supplementary material for: Inhibition of the Eukaryotic 80S Ribosome as a Potential Anticancer Therapy: A Structural Perspective
Source: Cancers (Basel). 2021 Aug 31;13(17):4392. doi: 10.3390/cancers13174392 (PMC8430933; doi:10.3390/cancers13174392)
Supplement: Supplementary file 1 [file cancers-13-04392-s001.zip › cancers-1356485-supplementary for XML/cancers-1356485-Supplementary_Table S1 for XML.docx]

Supplementary Materials: Inhibition of the Eukaryotic 80S Ribosome as a Potential Anticancer Therapy: A Structural Perspective

Simone Pellegrino, Salvatore Terrosu, Gulnara Yusupova and Marat Yusupov

**Table S1.** list of PDB codes for all the compounds discussed in the present review.

|  | **Organism ^1^** | **Compound Name** | **Ribosome Site** | **PDB code** |
| --- | --- | --- | --- | --- |
| 1 | *S. cerevisiae* | Cycloheximide | E site- 60S | 4U43 |
| 2 | *S. cerevisiae* | Lactamidomycin | E site- 60S | 4U4R |
| 3 | *S. cerevisiae* | C45 | E site- 60S | 6HHQ |
| 4 | *S. cerevisiae* | Chlorolissoclimide | E site- 60S | 5TBW |
| 5 | *S. cerevisiae* | Phyllanthoside | E site- 60S | 4U4Z |
| 6 | *S. cerevisiae* | Anisomycin | PTC (A-site) | 4U3M |
| 7 | *S. cerevisiae* | Lycorine | PTC (A-site) | 4U4U |
| 8 | *S. cerevisiae* | Narciclasine | PTC (A-site) | 4U51 |
| 9 | *S. cerevisiae* | Homoharringtonine | PTC (A-site) | 4U4Q |
| 10 | *S. cerevisiae* | T-2 toxin | PTC (A-site) | 4U6F |
| 11 | *S. cerevisiae* | Haemanthamine | PTC (A-site) | 5ON6 |
| 12 | *S. cerevisiae* | Agelastatin A | PTC (A-site) | 5MEI |
| 13 | *S. cerevisiae* | Nagilactone C | PTC (A-site) | 4U52 |
| 14 | *S. cerevisiae* | Deoxynivalenol | PTC (A-site) | 4U53 |
| 15 | *S. cerevisiae* | Verrucarin A | PTC (A-site) | 4U50 |
| 16 | *S. cerevisiae* | Blasticidin S | PTC (P-site) | 4U56 |
| 17 | *S. cerevisiae* | Amicoumacin A | mRNA path | 5I4L |
| 18 | *S. cerevisiae* | Cryptopleurine | mRNA path | 4U55 |
| 19 | *S. cerevisiae* | Pactamycin | mRNA path | 4U4Y |
| 20 | *S. cerevisiae* | Edeine | mRNA path | 4U4N |
| 21 | *S. cerevisiae* | Geneticin (G418) | Decoding centre | 5NDG |
| 23 | *S. cerevisiae* | Gentamicin | Decoding centre | 5OBM |
| 24 | *S. cerevisiae* | Paromomycin | Decoding centre | 5NDV |
| 25 | *S. cerevisiae* | TC007 | Decoding centre | 5NDW |
| 26 | *H. sapiens* | PF846 | Peptide Exit Tunnel | 6XA1 |
| 27 | *H. sapiens* | Hygromycin B | Decoding centre | 6EK0 |
| 28 | *T. thermophilus* |  |  | 4V5D |

**^1^** Organism from which ribosomes were purified and used for structural investigations.
